# Leptospirosis in American Samoa – Estimating and Mapping Risk Using Environmental Data

**DOI:** 10.1371/journal.pntd.0001669

**Published:** 2012-05-29

**Authors:** Colleen L. Lau, Archie C. A. Clements, Chris Skelly, Annette J. Dobson, Lee D. Smythe, Philip Weinstein

**Affiliations:** 1 School of Population Health, The University of Queensland, Herston, Australia; 2 WHO/FAO/OIE Collaborating Centre for Reference and Research on Leptospirosis, Coopers Plains, Australia; 3 Barbara Hardy Institute, University of South Australia, Adelaide, Australia; University of California San Diego School of Medicine, United States of America

## Abstract

**Background:**

The recent emergence of leptospirosis has been linked to many environmental drivers of disease transmission. Accurate epidemiological data are lacking because of under-diagnosis, poor laboratory capacity, and inadequate surveillance. Predictive risk maps have been produced for many diseases to identify high-risk areas for infection and guide allocation of public health resources, and are particularly useful where disease surveillance is poor. To date, no predictive risk maps have been produced for leptospirosis. The objectives of this study were to estimate leptospirosis seroprevalence at geographic locations based on environmental factors, produce a predictive disease risk map for American Samoa, and assess the accuracy of the maps in predicting infection risk.

**Methodology and Principal Findings:**

Data on seroprevalence and risk factors were obtained from a recent study of leptospirosis in American Samoa. Data on environmental variables were obtained from local sources, and included rainfall, altitude, vegetation, soil type, and location of backyard piggeries. Multivariable logistic regression was performed to investigate associations between seropositivity and risk factors. Using the multivariable models, seroprevalence at geographic locations was predicted based on environmental variables. Goodness of fit of models was measured using area under the curve of the receiver operating characteristic, and the percentage of cases correctly classified as seropositive. Environmental predictors of seroprevalence included living below median altitude of a village, in agricultural areas, on clay soil, and higher density of piggeries above the house. Models had acceptable goodness of fit, and correctly classified ∼84% of cases.

**Conclusions and Significance:**

Environmental variables could be used to identify high-risk areas for leptospirosis. Environmental monitoring could potentially be a valuable strategy for leptospirosis control, and allow us to move from disease surveillance to environmental health hazard surveillance as a more cost-effective tool for directing public health interventions.

## Author Summary

Leptospirosis is the most common bacterial infection transmitted from animals to humans. Infected animals excrete the bacteria in their urine, and humans can become infected through contact with animals or a contaminated environment such as water and soil. Environmental factors are important in determining the risk of human infection, and differ between ecological settings. The wide range of risk factors include high rainfall and flooding; poor sanitation and hygiene; urbanisation and overcrowding; contact with animals (including rodents, livestock, pets, and wildlife); outdoor recreation and ecotourism; and environmental degradation. Predictive risk maps have been produced for many infectious diseases to identify high-risk areas for transmission and guide allocation of public health resources. Maps are particularly useful where disease surveillance and epidemiological data are poor. The objectives of this study were to estimate leptospirosis seroprevalence at geographic locations based on environmental factors, produce a predictive disease risk map for American Samoa, and assess the accuracy of the maps in predicting infection risk. This study demonstrated the value of geographic information systems and disease mapping for identifying environmental risk factors for leptospirosis, and enhancing our understanding of disease transmission. Similar principles could be used to investigate the epidemiology of leptospirosis in other areas.

## Introduction

Leptospirosis is the most common bacterial zoonosis around the world [1], and its emergence has been linked to many environmental and ecological drivers of disease transmission. Varying environmental health hazards operate in different settings, and include climate, flooding, land use, urbanisation, poor sanitation (e.g. urban slums), international trade and travel, environmental degradation, and loss of biodiversity [2]–[11]. Accurate data on disease incidence and outbreaks are lacking in many parts of the world because of the combination of poor awareness of the disease, low clinical suspicion, varied clinical presentations leading to misdiagnosis, and the lack of laboratory facilities to confirm diagnoses [12].

Reported incidence of leptospirosis in the Pacific Islands is high compared to other parts of the world [13]–[16], and outbreaks have been reported recently [17]–[19]. However, most Pacific Islands do not have accurate epidemiological data on leptospirosis, making it difficult to quantify the importance of risk factors or predict outbreaks.

Environmental data, geographic information systems (GIS), spatial statistical analysis, and predictive risk maps have been used for the investigation and management of a range of infectious diseases including schistosomiasis [20], malaria [21]–[24], trachoma [25] and Rift Valley fever [26]. These maps identify geographic areas with high disease prevalence and/or risk of outbreaks, and are useful for guiding allocation of scarce public health resources and interventions. Such maps are particularly useful where disease surveillance data are poor or lacking. To date, no predictive risk maps have been produced for leptospirosis.

This study follows our reports on a seroprevelance study of leptospirosis in American Samoa in 2010 [27], [28]. The overall seroprevalence was 15.5% for the five islands surveyed, and 16.2% on the main island of Tutuila where over 95% of the population lived. The three most common reactive serovars on Tutuila were *L. interrogans* serovars Hebdomadis, LT 751, and LT 1163, with seroprevalences of 10%, 4.3%, and 3.5% respectively. Significant risk factors for seropositivity included male gender, outdoor occupation, low income, lack of knowledge about leptospirosis, living below median altitude of the village, and high density of piggeries around the home [27]. The three predominant serovars differed in their geographic distribution [28], and were associated with different risk factors [27].

This study further examined potential environmental health hazards for disease transmission using environmental data and geospatial analysis. The objectives of this study were to estimate leptospirosis seroprevalence at geographic locations based on environmental factors, produce a predictive disease risk map for American Samoa, and assess the accuracy of the maps in predicting infection risk. The results demonstrated that environmental health hazard surveillance could be a valuable strategy for identifying high-risk areas for disease transmission, and potentially be used as an adjunct or alternative to disease surveillance for targeting public health interventions for leptospirosis [29].

## Methods

### Data

#### Seroprevalence study

The data for this study were obtained from a seroprevalence study conducted in American Samoa from May to July 2010. Blood samples were collected from 807 participants on five islands, and questionnaire data were used to explore associations between seropositivity and individual-level risk factors (demographics, and exposures at home, work, and during recreation). Geo-referenced environmental data were used to explore associations between seropositivity and environmental factors around the home. The study design, study population, sampling technique, laboratory methods, and results have been described in detail in a recent report [27].

Ethics approvals were obtained from the American Samoa Institutional Review Board, the Medical Research Ethics Committee of The University of Queensland (2010000114), and Queensland Health Forensic and Scientific Services Human Ethics Committee (HREC/10/QFSS/1). Permission was also sought from the Department of Samoan Affairs and village chiefs before village visits. Verbal and written information on the study were provided in Samoan and/or English according to the participants' preference, and written informed consent was obtained from all participants. All data were de-identified prior to analyses.

**Figure 1:**
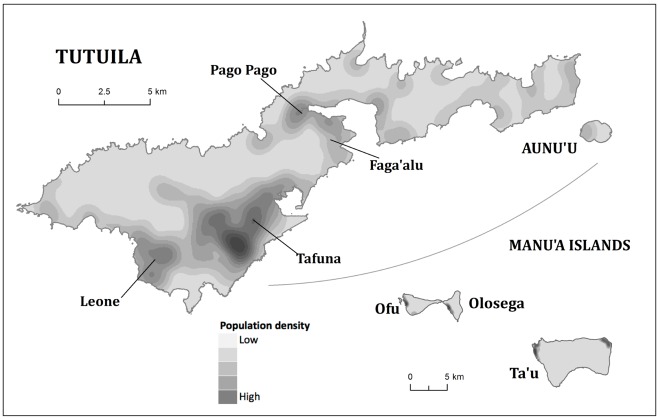
Population distribution on the islands of American Samoa, 2010 [28].

For this study of disease risk mapping, only data from the main island of Tutuila were included. There were 721 participants from 592 households, and 84% of households had only one participant. The populations and inhabited areas on the other islands were too small for geospatial analysis to be meaningful. Figure 1 shows the population distribution on Tutuila and the other islands of American Samoa.

#### Environmental data

Participants were geo-located to their place of residence, and all environmental variables were assessed at the household level. Data were collated, stored, linked and mapped using the GIS software, ArcMap v10.0 (Environmental Systems Research Institute, Redlands, CA).

Environmental data on coastline, rainfall, streams, flooding risk (as determined by a flood insurance risk map), location of houses and other buildings, and soil type were obtained from the American Samoa Geographic Information Systems User Group [30]. Altitudes of houses and piggeries were obtained using a digital elevation model [31] of American Samoa, and houses were classified into those above or below the median altitude of the village. Vegetation type was obtained from a recent vegetation mapping project [32], and classified into agricultural (vegetated land used for commercial production), urban built-up (impervious urban surfaces such as houses and paved roads), urban cultivated (vegetated areas within a general urban boundary, including fruit trees around homes, gardens, parks, sports fields, and lawns), or other vegetation types (including forests, scrubs, marshes, swamps, mangroves, and beaches). Geo-referenced data on the location of piggeries were provided by the American Samoa Environmental Protection Agency (ASEPA) [33]. Using counts of piggeries within 250 m buffers of houses and the relative altitude of houses and piggeries, an aggregate variable “number of piggeries within 250 m and above the house” was calculated for all house locations. Additional environmental variables calculated or extracted from these sources included density of houses around sampled locations (measured by number of houses within 250 m buffers of sampled houses), slope, distance to the closest stream, distance to the closest forested area, and distance to the closest coast.

The seroprevalence study also collected questionnaire data on a number of household-level environmental variables. Some variables were associated with specific serovars and were discussed in detail in a previous paper [27], but none were found to be significantly associated with overall seropositivity and therefore were not used for predictive risk mapping in this study. Variables assessed in the questionnaire included owning animals (dogs, cats, pigs, chickens), bats around the home, sighting or touching rats, working with animals, exposure to flooding, having an indoor toilet and/or shower, bathing in streams, growing vegetables and/or fruit trees around the home, type of sewage system, and the availability of garbage collection services [27].

### Statistical Analysis

#### Spatial cluster detection

SaTScan software [34] was used to identify spatial clustering of seropositive and seronegative cases. Kulldorff's scan statistic was calculated by using a moving circular window to test whether cases were distributed randomly over space, and to identify both high and low seroprevalence clusters. The statistic was set to include a maximum of 50% of the data. A Bernoulli model was used because the outcome variable was dichotomous (seropositive or seronegative). Statistically significant clusters were identified using p<0.05. SaTScan analyses were performed for all serovars, and separately for each of the three most commonly identified serovars.

#### Logistic Regression Analysis

Logistic regression for grouped data was used to take into consideration that some households (16%) had multiple participants. Multivariable logistic regression analysis was performed to investigate the association between risk factors and seropositivity (for all serovars). Univariate logistic regression analysis was initially performed for all variables, and variables with p<0.1 were retained in a multivariable model. Using a backwards stepwise approach, variables with p<0.05 on multivariable analysis were retained in the final model. STATA v11.1 software (StataCorp, College Station, Texas) was used for statistical analyses.

### Two logistic regression models were developed

- Model A: included environmental risk factors only
- Model B: incorporated both individual-level and environmental risk factors

Residuals of multivariable models were explored for spatial autocorrelation using semi-variograms. This was performed in the R statistical software package, version 2.9.0 (The R Foundation for Statistical Computing), using the geoR package.

#### Model goodness of fit

Statistical measures used to assess and compare the goodness of fit of the two models included Akaike information criterion (AIC); measures of in-sample predictive ability using area under the curve of the receiver operating characteristic (AUC); and the percentage of cases that were correctly classified as seropositive or seronegative using the models.

#### Model validation

The models were cross-validated by measuring out-of-sample predictive ability of the model. The dataset was randomly divided into four subgroups of equal numbers. Multivariable models were developed with data from three subgroups, and used to predict seroprevalence for the fourth group. This procedure was repeated four times by using different combinations of three subgroups to develop the multivariable model, and predicting seroprevalence in the remaining subgroup. The accuracy of predictions of each model was validated by comparing the predicted occurrence with observed occurrence of seropositive cases, using a seroprevalence threshold of 50% to predict seropositive cases. The discriminatory performance of each model was measured using AUC, and the percentage of seropositive cases that were correctly classified. An AUC of 0.7 was deemed to indicate an adequate predictive ability of the model [35], [36].

#### Predicting spatial variation in seroprevalence

To eliminate uninhabited areas of the island from analyses, areas further than 250 m from existing buildings were excluded. Using the multivariable logistic regression models described above, coefficients of covariates were used to predict seroprevalence for the locations of the nodes of a 50 m×50 m grid overlaid on a map of Tutuila. For both models, predicted seroprevalence varied spatially according to the values of the environmental covariates. For Model B, seroprevalence was predicted for different combinations of the individual-level covariates, including: i) the combination of individual-level covariates that generated the highest risk (i.e. males, outdoor workers, and people who had no knowledge of leptospirosis), and ii) the combination of individual-level covariates that generated the lowest risk (i.e. females, indoor workers, and people who had knowledge of leptospirosis). Because the effects of the individual-level covariates are constant through space, this resulted in maps with high and low mean predicted seroprevalence, but similar spatial patterns in seroprevalence relative to the mean.

## Results

### Spatial clustering

**Figure 2:**
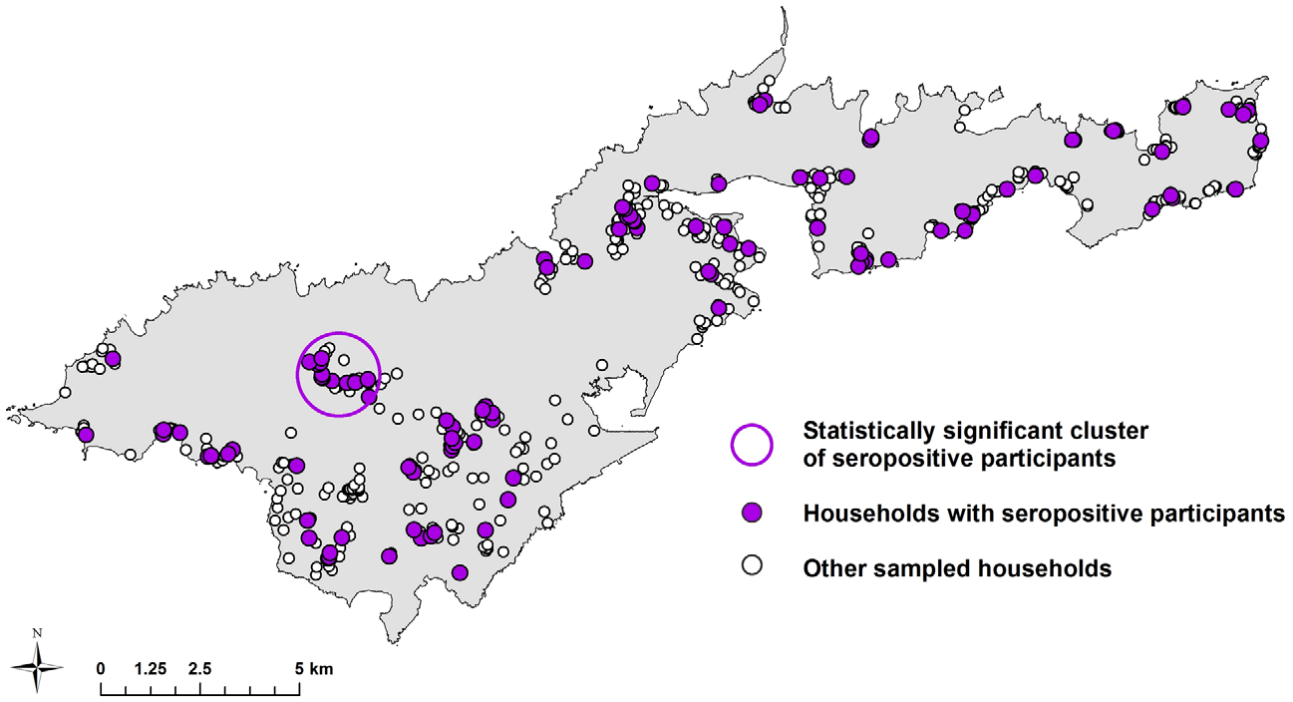
Statistically significant cluster of participants seropositive for leptospirosis (all serovars). Cluster included 8 positive cases out of 10 sampled (relative risk 5.34, p = 0.022). Calculated using Kulldorff's spatial scan statistic [34].

**Figure 3:**
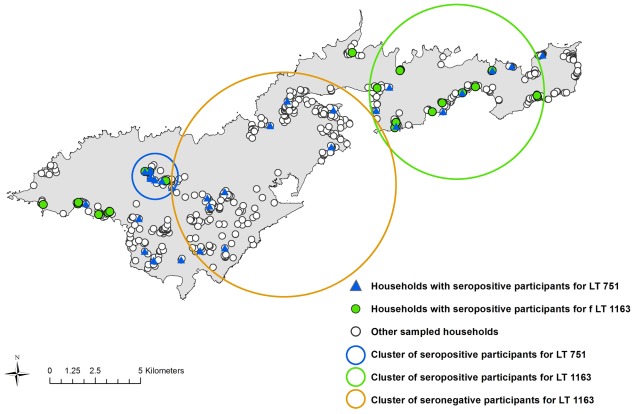
Statistically significant clusters of participants seropositive and seronegative for leptospirosis (specific serovars). Seropositive cluster for LT 751 included 7 positive cases out of 13 sampled (RR 16.24, p = 0.0032); seropositive cluster for LT 1163 included 14 positive cases out of 130 sampled (RR 5.94, p = 0.02); seronegative cluster for LT 1163 included 0 positives out of 290 sampled (RR 0, p = 0.0016). Calculated using Kulldorff's spatial scan statistic [34]. RR = relative risk.

Four statistically significant clusters (three seropositive and one seronegative) were identified. When scanning all serovars, one seropositive cluster was identified in an area where over 50% of participants were seropositive. When scanning for individual serovars, two seropositive clusters were identified (one each for LT 751 and LT 1163), and a seronegative cluster was identified for LT 1163 in an area where none of the 290 participants tested positive for this serovar. Statistical details of the clusters are shown in Table 1, and locations of the clusters are shown in Figures 2 and 3.

**Table 1:** Statistically significant clusters of participants seropositive and seronegative for leptospirosis in American Samoa, 2010.

	SEROPOSITIVE CLUSTERS			SERONEGATIVE CLUSTER
	All serovars	LT 751^a^	LT 1163^b^	LT 1163^b^
**Relative risk**	5.34	16.24	5.94	0
**P value**	0.022	0.00032	0.02	0.0016
**Number of participants**	10	13	130	290
**Number of seropositive cases**	8	7	14	0

a *Leptospira interrogans* serovar LT 751.

b *Leptospira interrogans* serovar LT 1163.

### Multivariable models and goodness of fit

Statistically significant covariates on multivariable analyses and measures of goodness of fit for models A and B are shown in Table 2. Four significant environmental risk factors were identified and included in Model A: (i) living below median altitude within a village, (ii) living on agricultural land, (iii) living on clay loam soils, and (iv) number of piggeries located within 250 m and above the house. Additionally, three individual-level risk factors were identified: (i) male gender, (ii) occupational risk (outdoor workers and fish cleaners), and (iii) lack of knowledge about leptospirosis. Model B incorporated both environmental and individual-level risk factors. No significant residual spatial autocorrelation was found, suggesting that spatial clustering was largely explained by the covariates included in the models.

**Table 2:** Multivariable logistic regression models of leptospirosis seropositivity in American Samoa, 2010.

SIGNIFICANT RISK FACTORS	MODEL AOdds Ratio (95% CI)	MODEL BOdds Ratio (95% CI)
**QUESTIONNAIRE VARIABLES:**		
**Male** a	**-**	**2.77 (1.74–4.42)**
**Occupational groups:**		
• Indoor	-	1
• Outdoor (including fish cleaners)	**-**	**2.77 (1.40–5.49)**
• Mixed Indoor/Outdoor	-	1.14 (0.46–2.87)
• Unemployed	-	1.59 (0.85–2.98)
**Heard of leptospirosis** b	**-**	**0.60 (0.38–0.96)**
**ENVIRONMENTAL VARIABLES:**		
**House below median altitude of village:**		
• No	1	1
• Yes	1.47 (0.96–2.27)	**1.58 (1.00–2.49)**
**Vegetation type:**		
• Urban built up	1	1
• Urban cultivated	1.22 (0.74–1.99)	1.13 (0.67–1.88)
• Agricultural	**2.33 (1.28–4.23)**	**2.09 (1.12–3.89)**
• Other	2.21 (0.69–7.07)	1.66 (0.49–5.61)
**Soil type:**		
• Clay	1	1
• Clay loams	**3.11 (1.27–7.61)**	**2.72 (1.08–6.85)**
• Urban	2.04 (0.81–5.10)	1.86 (0.72–4.78)
• Other	2.20 (0.79–6.14)	2.09 (0.73–5.98)
**Piggeries within 250 m and above house** c **:**	**1.16 (1.07–1.26)**	**1.15 (1.05–1.26)**
**MEASURES OF MODEL GOODNESS OF FIT:**		
Akaike information criterion (AIC)	624.62	585.83
Area under the curve of ROC (AUC)	0.65 (0.60–0.71)	0.74 (0.69–0.79)
% of cases correctly classified	84.05%	84.43%

Model A based on environmental risk factors. Model B based on a combination of individual-level and environmental risk factors.

a Compared to females.

b Compared to people who had never heard of leptospirosis.

c Continuous variable. Odds ratio reflects increase in risk for each extra piggery within 250 m and at a higher altitude than the house.

Statistically significant odds ratios highlighted in bold.

### Model validation

Using the four subsets of the models for validation, the average AUC was 0.63 for Model A and 0.70 for Model B. An average of 84.05% and 83.11% of cases in the fourth subset were correctly classified in Model A and Model B respectively, indicating that model had acceptable predictive performance.

### Spatial variation in predicted seroprevalence

**Figure 4:**
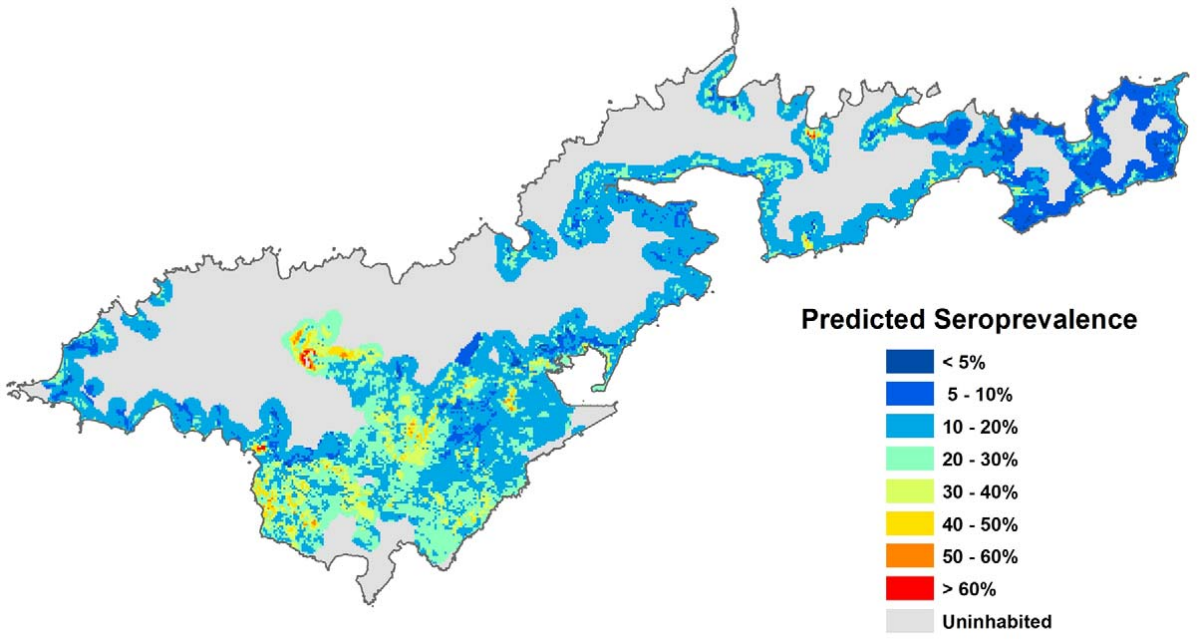
Predicted leptospirosis seroprevalence based on environmental variables. Predicted values were calculated using Model A, based on four environmental variables (altitude, piggeries, vegetation, and soil type).

**Figure 5 pntd-0001669-g005:**
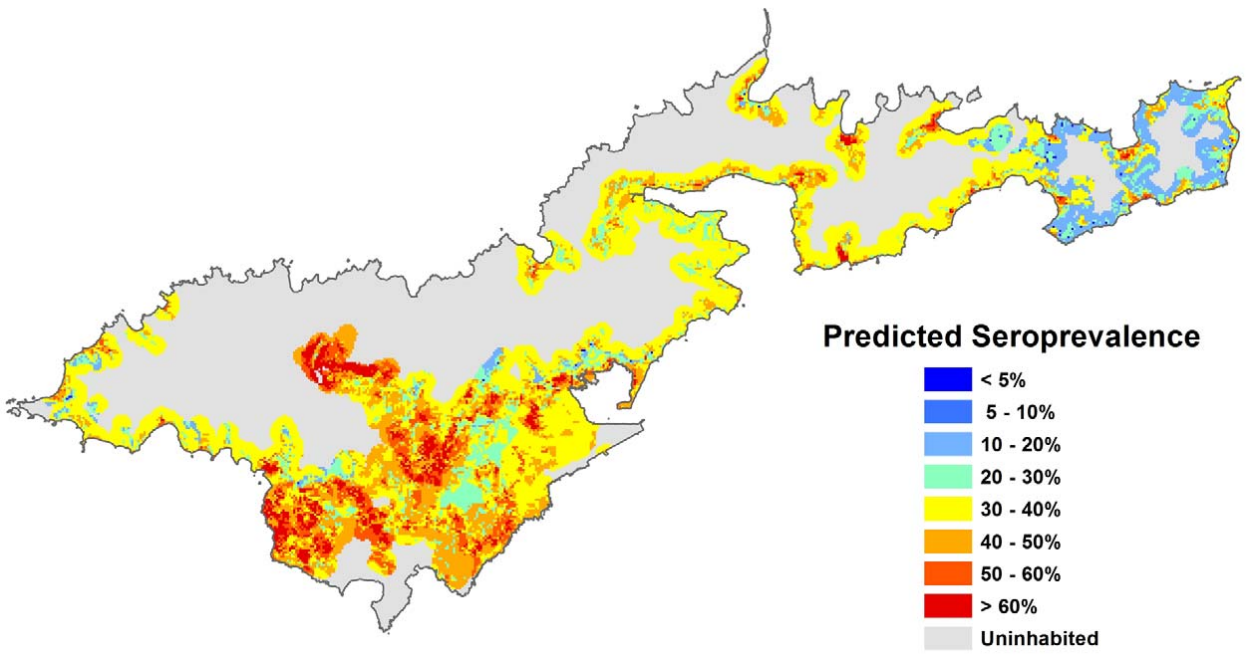
Predicted leptospirosis seroprevalence based on environmental variables and individual-level variables associated with the highest risk. Predicted values were calculated using Model B, based on four environmental variables (altitude, piggeries, vegetation, and soil type), and three individual-level variables associated with the highest risk (males, outdoor workers, and no knowledge of leptospirosis).

**Figure 6:**
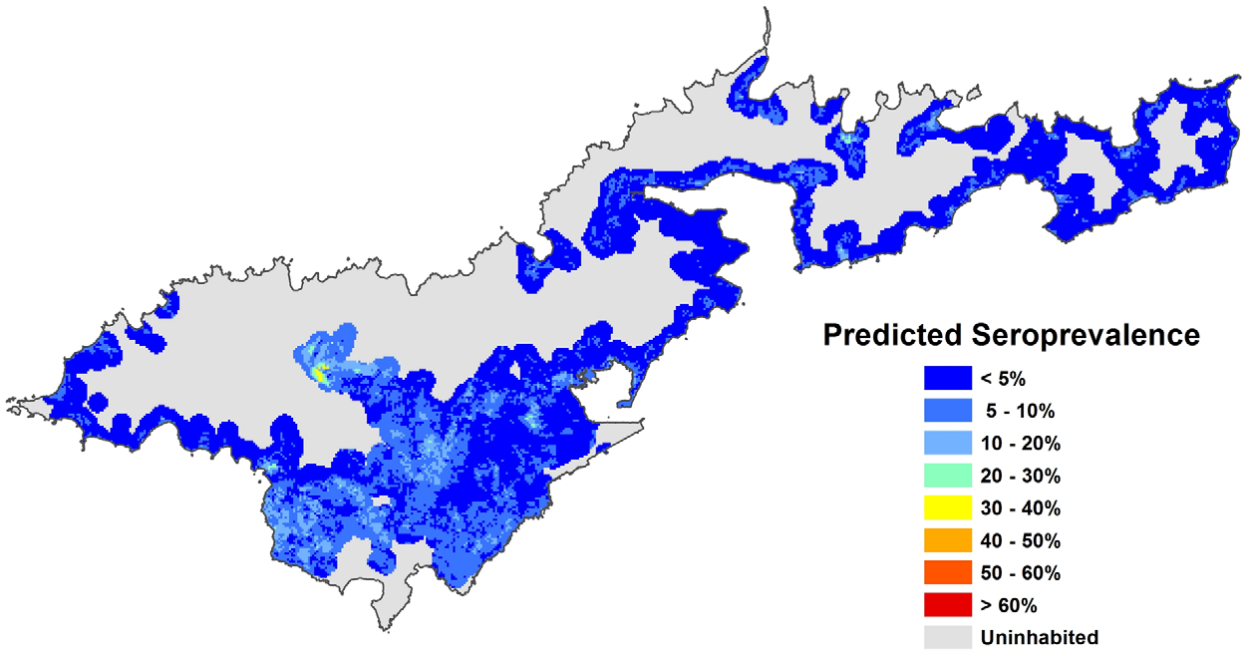
Predicted leptospirosis seroprevalence based on environmental variables and individual-level variables associated with the lowest risk. Predicted values were calculated using Model B, based on four environmental variables (altitude, piggeries, vegetation, and soil type), and three individual-level variables associated with the lowest risk (females, indoor workers, and knowledge of leptospirosis).

The following seroprevalence prediction maps were generated:

- Using Model A, based on environmental risk factors only (Figure 4)
- Using Model B, based on environmental risk factors and the combination of individual risk factors that generated the HIGHEST risk, i.e. males, outdoor workers/fish cleaners, and people who had never heard of leptospirosis (Figure 5)
- Using Model B, based on environmental risk factors and the combination of individual risk factors that generated the LOWEST risk, i.e. females, indoor workers, and people who had heard of leptospirosis (Figure 6)

### Number of houses with different levels of predicted seroprevalence

**Figure 7:**
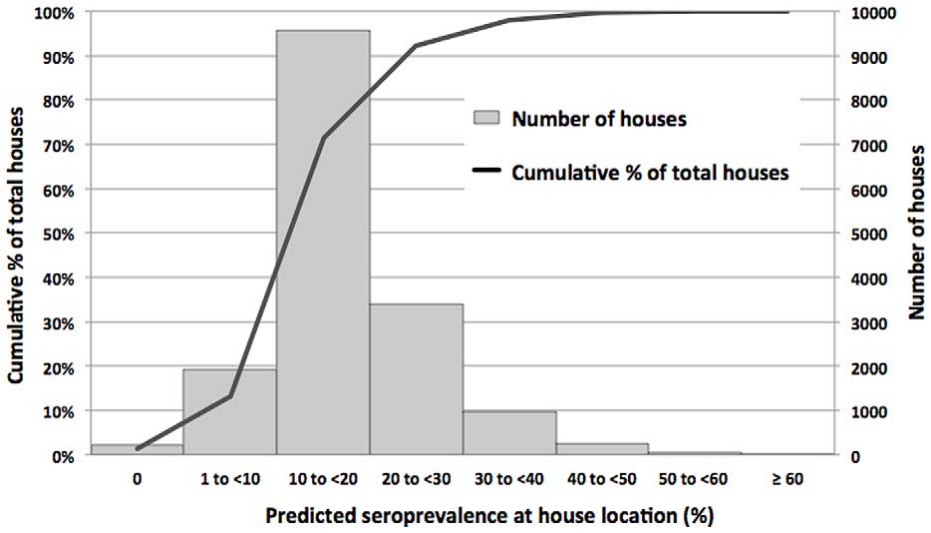
Number of houses with different levels of predicted leptospirosis seroprevalence. Values were calculated by overlaying a map of house locations over the risk prediction map in Figure 4.

Based on Model A and the map in Figure 4, the predicted seroprevalence was extracted for all houses on Tutuila to provide information on the proportion of the population exposed to different levels of risk. Figure 7 shows that based on environmental covariates alone, 58.3% of houses had a predicted seroprevalence of 10 to 20%, and 90.9% of houses had a predicted seroprevalence of 1 to 30%.

### Seroprevalence prediction chart

**Figure 8:**
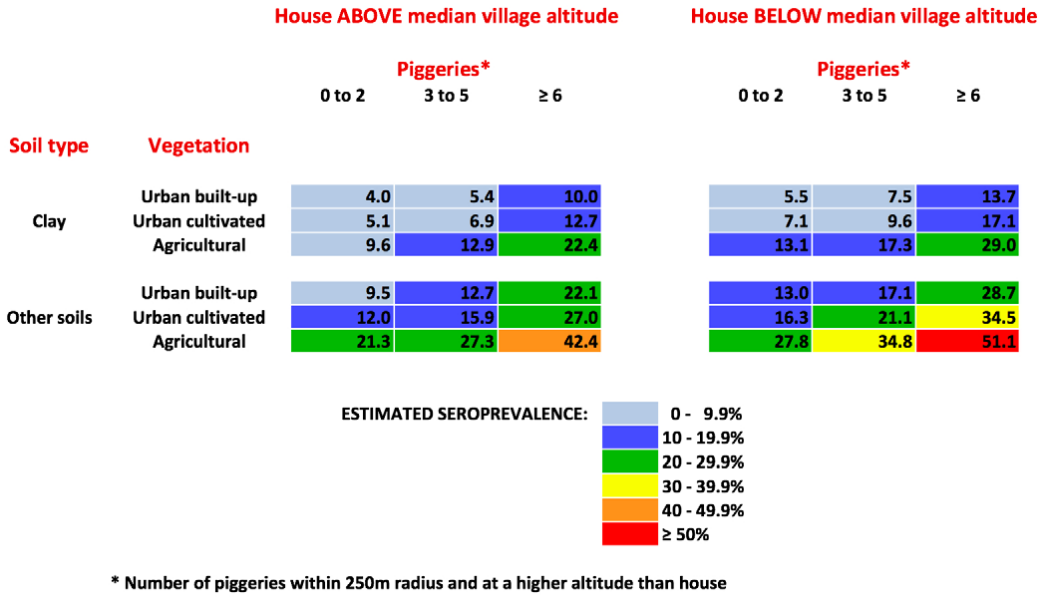
Seroprevalence prediction chart based environmental risk factors at home. The chart shows the combined effects of four environmental variables (altitude, piggeries, soil type, vegetation type) in determining overall risk.

A seroprevalence prediction chart was generated based on the four statistically significant environmental variables (“number of piggeries within 250 m and above the house”, altitude, vegetation type, soil type). Figure 8 shows that individuals who have two or fewer piggeries within 250 m and above their home, live above the median altitude of their village, in urban built-up areas, and on clay soil have a predicted seroprevalence of 4%; whereas those who have more than six piggeries within 250 m and above their home, live below the median altitude of their village, in agricultural areas, and on non-clay soils have a predicted seroprevalence of 51.1%.

## Discussion

In American Samoa, seropositivity to leptospirosis was associated with environmental as well as individual-level factors. Significant household-level environmental hazards included those related to the natural environment (altitude and soil type) as well as anthropogenic activities (agriculture and piggeries). Results of this study corroborate findings from other studies that the household environment is an important determinant of leptospirosis infection risk [8], [37]–[39].

Living below the median altitude of a village was associated with seropositivity, and was likely to be related to greater exposure to run-off from higher parts of the village, carrying pathogens including leptospires. Lower altitudes would also be more prone to flooding. Living on clay soil was associated with a lower risk of infection. Clay soils absorb water poorly and would allow rain to run off rapidly. In contrast, clay loams and other soils absorb and hold water (and leptospires) for longer periods of time, and could thereby increase the exposure risk for those who lived in these areas. Soil temperature and acidity could also potentially affect leptospire survival in the environment [40], but there were insufficient data on soil characteristics to explore this explanation. Living in agricultural areas was associated with seropositivity, and was likely to be related to farming activities and exposure to animals.

The large number of pigs and backyard piggeries in AS have previously been implicated in leptospirosis transmission [41]. In 2010, there were approximately 430 backyard piggeries housing 3500 pigs (ASEPA, pers. comm), and efforts have been made to control and regulate their numbers and design [33]. In this study, piggery density was measured by counting the number of piggeries located within 250 m of houses and at a higher altitude. Similar analysis using greater buffer distances of 350 m, 500 m, 750 m, and 1000 m also produced statistically significantly results, but the strength of association decreased with increasing buffer distances. Larger buffers often included other valleys and watersheds, and were therefore deemed inappropriate. Analysis with buffer distances of 100 m did not produce any significant results, probably because there were few piggeries located within 100 m of houses. A buffer distance of 250 m was chosen for analysis because it provided the best prediction of seropositivity.

The number of piggeries located at a lower altitude than houses was not associated with seropositivity for any of the above buffer distances, suggesting that drainage of refuse downhill from piggeries is an important source of infection. The association between piggeries and leptospirosis seropositivity was potentially epidemiological rather than causal, and the true source of infection could have been the rodents around piggeries rather than the pigs. Despite this, proper management of piggery waste should still reduce the risk of exposure for people living downhill from piggeries. Further studies involving the collection of samples from animals would be required to determine which animal species are the primary carriers of leptospiral serovars responsible for human infection.

This study showed that both individual-level and environmental risk factors combined to determine the overall risk of human leptospirosis in American Samoa. Effective public health interventions would therefore need to include strategies to reduce individual risk as well as environmental exposures [27]. Strategies to reduce exposure risk in individuals include improvements in occupational health and safety (e.g. by wearing protective clothing) and community knowledge about leptospirosis. At the community level, proper management of piggeries and building piggeries further away from homes could reduce exposure to piggery waste. Altitude and soil type were associated with infection risk and as discussed above, are likely to be related to the risk of flooding. In the Pacific, flooding is predicted to occur more frequently with global climate change as a result of more intense rainfall and cyclones. It would therefore be important to reduce flooding risk by improving drainage and keeping drains clear of garbage and debris. Communities should also be advised to avoid floodwaters.

In contrast to many other studies, rainfall and flooding risk were not statistically significantly associated with seropositivity in this study. American Samoa is one of the wettest inhabited places in the world with an average annual rainfall of more than 3000 m, and it was therefore possible that all areas of the island were at high risk in this environment. The flood risk map available was produced to identify areas susceptible to severe damage for insurance purposes, and was possibly a poor indicator of overall flooding risk and exposure [27].

The questionnaire used in the seroprevalence study explored many household-level environmental exposures known to be associated with leptospirosis infection, but none were found to be associated with overall seropositivity [27]. However, some of the exposures were widespread, making it difficult to determine their effect on infection risk. For example, 65% of participants reported sighting rats or mice at home and 75% reported bats around the home. Water and sanitation services were also very similar for all participants. Ninety-six % had piped water, 90% had an indoor toilet, 89% had an indoor shower, 87% had garbage collection services, and only one person did not have a sewage system (mains sewage or septic tank) at home. Furthermore, owning animals was not associated with seropositivity possibly because people in American Samoa were often exposed to animals even though they were not the owners. In this study, 67% of participants reported owning dogs but almost the entire population would be exposed to the large numbers of unrestrained dogs responsible for one of the highest reported incidence of dog bites in the world [42]. Similarly owning pigs was not associated with seropositivity, but geospatial analysis described in this study showed that piggeries around the home were associated with infection risk. In this study, geo-referenced data were more useful than questionnaire data for identifying environmental risk factors.

The maps in Figures 2 and 3 show that there were geographic areas with significant clusters of seropositive and seronegative cases. Clusters varied between serovars, suggesting different environmental and ecological drivers of disease transmission. In a recent related paper that explored the ecological drivers of leptospiral serovar emergence in American Samoa, serovar LT 1163 was found to be completely absent in the more highly populated parts of the island [28]. Figure 3 shows that serovar LT 1163 was only found in the less populated parts of the island, and the seronegative cluster corresponds to the most densely populated area. In this study of predictive risk mapping, all serovars were combined in the analysis and there was no significant association between population density and overall seroprevalence. Serovar-specific predictive risk maps could be produced if future studies collected larger datasets, and might be more accurate than maps that include all serovars.

The map in Figure 4 shows the variation in predicted seroprevalence based on environmental health hazards alone. Figures 5 and 6 show the predicted seroprevalence for the highest and lowest risk individuals living in different parts of the island, and that infection risk could be significantly increased by individual-level factors. The statistically significant positive cluster for all serovars on SaTScan (Figure 2) corresponds accurately to an area of predicted high seroprevalence on the risk maps in Figures 4 to [Fig pntd-0001669-g005] 6. This area was situated on a steep hill, where there were large numbers of piggeries located behind and above houses.

Figure 7 shows that the majority of houses in Tutuila were located in areas with a predicted seroprevalence of 10 to 20%, and was consistent with the observed population seroprevalence of 15.5% in our study in 2010. The number of houses in different risk categories was determined by the predicted seroprevalence as well as house density at each location, and provided an indication of overall disease burden. The seroprevalence prediction chart in Figure 8 shows the combined effects of the four environmental factors in determining infection risk, and provided a more accurate estimate of seroprevalence than individual risk factors alone, or a simple count of multiple risk factors.

The limitations of the seroprevalence study have been previously discussed [27]. The cross-sectional study design did not allow assessment of variations in disease incidence or risk factors over time. If available, long-term incidence data could provide additional information on the effect of seasons, rainfall, and natural disasters. There were also limitations to the use of serological tests for leptospirosis, and isolates of leptospires would be required to confirm the study findings. There were likely to be other environmental risk factors that were not explored in this study, and further research would be required to identify these hazards. The potential role that other animal species play in disease transmission should also be investigated. The accuracy of the models and risk maps were limited by the accuracy of environmental data, and changes in environmental variables over time. Prediction models and risk maps would need to be updated as environmental conditions change, and could be refined as additional information and data become available.

This study showed that it was possible to identify high-risk areas for leptospirosis based on environmental variables alone, and this approach could be useful for stratifying geographic locations according to risk, particularly when disease surveillance data are lacking. Environmental health hazard surveillance could therefore be a useful strategy for identifying high-risk locations for disease transmission, and should be considered as an alternative or complement to disease surveillance, which would generally be more costly, complex and difficult to manage. This strategy could potentially provide valuable information for targeting public health interventions and optimising resource allocation, particularly in areas with limited financial and public health resources, such as the Pacific Islands.

This study demonstrated the value of GIS and disease mapping for investigating the spatial distribution of leptospirosis infection, identifying geographic and environmental risk factors, and enhancing our understanding of disease transmission dynamics. The ability to accurately assess, predict, and map environmental drivers of disease transmission could also allow us to move from disease surveillance to environmental health hazard surveillance as a more cost-effective tool for directing public health interventions.

Although this study was specific to the cultural and environmental conditions in American Samoa, the principles might also be applicable to other endemic areas for leptospirosis, and the findings might be pertinent to other Pacific Islands with similar climate, ecosystems, animal reservoirs, lifestyle and culture.
